# Virulence Factors of the Periodontal Pathogens: Tools to Evade the Host Immune Response and Promote Carcinogenesis

**DOI:** 10.3390/microorganisms11010115

**Published:** 2023-01-01

**Authors:** Linah A. Shahoumi, Muhammad H. A. Saleh, Mohamed M. Meghil

**Affiliations:** 1 Department of Oral Biology and Diagnostic Sciences, The Dental College of Georgia at Augusta University, Augusta, GA 30912, USA; 2 Department of Periodontics and Oral Medicine, University of Michigan School of Dentistry, Ann Arbor, MI 48109, USA; 3 Department of Periodontics, The Dental College of Georgia at Augusta University, Augusta, GA 30912, USA

**Keywords:** *Aggregatibacter actinomycetemcomitans*, *Porphyromonas gingivalis*, *Fusobacterium nucleatum*, carcinogenesis, immune response

## Abstract

Periodontitis is the most common chronic, inflammatory oral disease that affects more than half of the population in the United States. The disease leads to destruction of the tooth-supporting tissue called periodontium, which ultimately results in tooth loss if uncured. The interaction between the periodontal microbiota and the host immune cells result in the induction of a non-protective host immune response that triggers host tissue destruction. Certain pathogens have been implicated periodontal disease formation that is triggered by a plethora of virulence factors. There is a collective evidence on the impact of periodontal disease progression on systemic health. Of particular interest, the role of the virulence factors of the periodontal pathogens in facilitating the evasion of the host immune cells and promotion of carcinogenesis has been the focus of many researchers. The aim of this review is to examine the influence of the periodontal pathogens *Aggregatibacter actinomycetemcomitans* (*A. actinomycetemcomitans*), *Porphyromonas gingivalis* (*P. gingivalis*), and *Fusobacterium nucleatum* (*F. nucleatum*) in the modulation of the intracellular signaling pathways of the host cells in order to evade the host immune response and interfere with normal host cell death and the role of their virulence factors in this regard.

## 1. Introduction

Despite being mostly preventable/manageable [1,2], periodontitis is universally prevalent, on a large scale [3,4]. Severe periodontitis has been declared the 6th most common human chronic disease [5], with an estimated prevalence of about 10–13% [5,6,7]. The collective direct costs for periodontal therapy in 2018 were estimated to be 2.5 billion Euros in Europe and 3.4 billion Dollars in the US. Further aggravating, the indirect costs (including loss of productivity, etc.) were estimated to be 21 and 14 times more in Europe and USA, respectively [8].

Periodontitis is a dental biofilm dysbiosis-induced, host-modified inflammatory disease that results in the breakdown of soft and hard periodontal tissues (attachment loss) [9] that is modified by various local and systemic risk drivers [10,11]. Periodontitis progression is accompanied by an exaggerated host immune response that has a significant pro-inflammatory disposition and was found to impact systemic health, and vice versa [12]. It is hence hardly surprising that several authors postulated that rebalancing metabolic cofactors help in mitigating the inflammatory cascade in periodontal disease [13].

Early onset periodontitis occurs in individuals < 20 years old and is characteristically associated with the bacterium *Aggregatibacter actinomycetemcomitans* (*A. actinomycetemcomitans*). Adult periodontitis has been linked to two bacteria, in particular, *Porphyromonas gingivalis* (*P. gingivalis*) *and Tannerella forsythia*. Both bacteria are found at higher levels in individuals with recent episodes of attachment loss and have been indicated to cause tissue destruction in animal models [14,15]. In particular, *P. gingivalis* is a highly adapted pathogen, equipped with many of putative virulence factors such as fimbriae and lectin-type adhesins, a polysaccharide capsule, lipopolysaccharides, potent proteinases, toxic products of metabolism, hemagglutinins, and numerous enzymes [16]. It is noteworthy that the terms early onset and adult periodontitis are no longer used according to the current classification of periodontal disease [17]. 

Many leukocytes have been hypothesized to play a role in periodontitis. These include neutrophils, lymphocytes, plasma cells, and monocytes. Additionally, resident gingival fibroblasts, periodontal ligament, and osteoblasts partake. Most involved cells produce chemokines [e.g., monocyte chemoattractant protein (MCP)-1], cytokines (e.g., interleukin (IL)-1, -6, and TNF), matrix metalloproteinases (e.g., MMP-1 and -8), and products of arachidonic acid metabolism (e.g., prostaglandin E2) [18]. Of note, *P. gingivalis*, was found to induce high levels of MCP-1 and IL-8 production in osteoblastic cells and leukocytes [19]. 

Numerous studies described the association of periodontitis with chronic systemic diseases, also revealing potential two-way relationships between periodontitis and overall health and systemic well-being [20,21,22]. The interconnectivity of systemic diseases including diabetes mellitus, cardiovascular diseases, metabolic syndrome, rheumatoid arthritis, Alzheimer’s disease, and different cancers with periodontitis is of chief research interest [23]. Such interconnectivity in some instances can be multi-modal/bi-directional. Proposed mechanisms of these relations include but are not limited to genetic factors, environmental factors (stress, smoking, and high-fat diet), bacteremia/viremia, and an altered host immune response [23]. 

These findings were evaluated in 2012 at a joint consensus from the European Federation of Periodontology and the American Association of Periodontology (EFP/AAP). Focusing on the most studied associations, the consensus concluded that periodontitis contributes to the systemic inflammatory responses, likely to act as a contributing factor in the pathophysiology of these morbidities. The consensus also highlighted that the role of systemic inflammation is a recurring theme in oral-systemic associations [24,25,26,27]. 

It has been hypothesized that periodontitis may increase the risk for cancer development (locally and distantly) due to its long-standing inflammatory nature [28]. Studies demonstrated the role of viruses such as Human Papillomavirus (HPV) and Epstein-Barr virus (EBV), which are detected in periodontal pockets, through activation of specific oncogenes (e.g., E6 and E7 for HPV) [29,30]. Again, *P*. *gingivalis* was proven to prevent cell apoptosis, thus favoring cancer initiation [31,32]. *P*. *gingivalis* and similar pathogens could be found in gingival cancers [31] and could also be linked to distant tumors [33]. 

As mentioned, periodontitis may provoke a significant surge in inflammatory markers, aggravating the inflammatory reaction. This results in the release of reactive oxygen species and other metabolites that could promote cancer initiation [34]. Besides, the inflammatory process and presence of cell-stimulating signals create an ideal milieu for cell proliferation and differentiation [34,35]. Such a mechanism could act both locally and at a distance [35]. 

Two relatively recent systematic reviews found a positive association between periodontitis and any type of cancer [28,36].

Hence the aim of this article is to review the knowledge of the immunomodulatory roles of the virulence factors of the periodontal pathogens *A. actinomycetemcomitans*, *P. gingivalis*, and *F. nucleatum.* In addition, we will examine the complex interplay between these virulence factors and the host cells, particularly evasion of the host immune response and promotion of carcinogenesis and discuss the role of the manipulation of intracellular signaling pathways in this regard.

## 2. Virulence Factors of Periodontal Pathogens and Their Association with Immune Escape and Carcinogenesis

### 2.1. A. actinomycetemcomitans

*A. actinomycetemcomitans* is a Gram-negative, facultative anaerobe, non-motile bacterium that is implicated in sever forms of periodontal disease that affects young individuals. Upon invading host cells via endocytosis, *A. actinomycetemcomitans* secretes phospholipase C to destroy membrane vesicles and release themselves into the cytoplasm. Six different *A. actinomycetemcomitans* serotypes have been identified based on LPS antigenicity. Of which, serotypes a-c are the most frequently isolated from Caucasians, Asians, Africans and Latin Americans, with serotype c being the most frequently isolated from periodontitis patients in general [37,38,39,40,41]. These different serotypes have been shown to induce differential DC and T-cell responses. DCs stimulated with serotype b produce high levels of IL-1β, IL-10, IL-12, IL-23, CCR5, and CCR6, relative to DCs stimulated with serotypes a and c [42]. In addition, DCs infected with serotype b have been shown to prime T cells to Th1 and Th17 phenotypes [42].

*A. actinomycetemcomitans* produces a variety of virulence factors such as adhesion proteins, lipopolysaccharides (LPS), and toxins to evade host innate defense mechanisms and promote carcinogenesis. Two toxins have been described for *A. actinomycetemcomitans*, leukotoxin (LtxA) and cytolethal distending toxins (CDT). 

LtxA is a member of the RTX (Repeats in ToXin) toxins, membrane-damaging proteins secreted by Gram-negative bacteria. RTX toxins are secreted across the bacterial envelope via the type I secretion system as a mode of export. RTX toxins are divided into three categories, broadly cytolytic RTX hemolysins, species-specific RTX leukotoxins, and large (>3200 amino acid residues), multifunctional, auto processing RTX toxins (MARTX) [43]. RTX leukotoxins are characterized by a cell type and species specificity, attributed to its cell-specific binding through the β2 integrin receptors, a family of receptors expressed on the surface of leukocytes and share a common β2 subunit, CD18, which is combined with either one of the unique α chains, α_L_ (CD11a), α_M_ (CD11b), α_X_ (CD11c), or α_D_ (CD11d) [44]. 

In addition to its crucial role in immune evasion, LtxA is suggested to be linked to the progression of periodontal disease through its effects on inducing pathogenic mechanisms in leukocytes [45]. LtxA induces humoral as well as cellular host immune response in periodontally diseased individuals [46]. The cytotoxic effects of LtxA against immune cells protects the bacterium from phagocytic killing. Exposure of neutrophils to LtxA results in activation of degranulation of neutrophils and extracellular release of proteolytic enzymes, such as elastase and matrix metalloproteases (MMPs) [47,48]. In addition, it has been reported that LtxA which results in increased production of pro-inflammatory cytokines (i.e., IL-1β and IL-18) and activation of the inflammasome complex through its action on macrophages [49]. Furthermore, *A. actinomycetemcomitans* expresses an outer membrane protein, called bacterial interleukin receptor I (BilRI), which binds to host cytokines, including IL-1β [50]. BilRI was also shown to play a role in the internalization of IL-1β by *A. actinomycetemcomitans* and that deletion of the *bilRI* gene results in significant decrease in internalization of IL-1β [50]. Recently, a study has shown that LtxA can hijack the endocytic trafficking pathways in lymphocytes via LtxA/LFA-1 internalization complex without damaging the plasma cell membrane [51]. LtxA follows the lysosomal degradation pathway in colocalization with LFA-1 and dissociates from it in the low PH of the endosomal environment, causing rupture of the lysosomal membrane at the terminal step of the lysosomal degradation [51], resulting in protecting the bacterium from phagocytic killing [52]. Collectively, the ability of LtxA to create a proteolytic environment that results in degradation of the host’s immunoproteins, internalize pro-inflammatory cytokines, degrade the host’s lysosomal and endosomal vesicles, and kill immune cells may all contribute to hijacking the host immune system by *A. actinomycetemcomitans* and survival within it. 

Cytolethal distending toxins (CDT) is a bacterial toxin produced by several Gram-negative pathogenic bacteria. CDT was first identified in the 1980s in some *Escherichia coli* strains, *Shigella* and *Campylobacter* species [53,54,55]. Later, CDT was found to be produced by several other Gram-positive bacteria, including *A. actinomycetemcomitans* [56,57]. CDT is a heterotrimeric toxin composed of three subunits, CdtA, CdtB and CdtC [58]. CDT is considered AB2 trimer toxin with two regulatory subunits (CdtA and CdtC) responsible for the transport of the thirds, enzymatically active subunit CdtB. CtdB functions as a DNase causing DNA damage which triggers activation of the G2/M checkpoint, resulting in induction of cell cycle arrest followed by apoptotic cell death [59,60]. While CdtA and CdtC play a role in anchoring CdtB on host cell membrane, CdtC is considered to be a chaperone for CdtB transfer. CDT binds to the host cell membrane via CdtA and CdtC, a step that depends on the presence of intact lipid rafts. Upon entering the host cell by dynamin-dependent endocytosis, cdtB translocates to the endoplasmic reticulum and subsequently to the nucleus [61]. The potent DNase activity of CDT induces DNA damage by causing single-strand breaks (SSB) and stalling of the replication forks, which ultimately leads to double-strand breaks (DSB), causing replication stress response and cell cycle arrest or even apoptotic death via CDT-mediated apoptosis [62,63]. DSB is detected via The Mre11-Rad50-Nbs1 (MRN) complex. Nbs1 recruits ATM (ataxia-telangiectasia mutated) kinase to damaged DNA, where it undergoes autophosphorylation [64]. ATM-dependent cell cycle arrest involves phosphorylation of p53 by Activated ATM, resulting in induction of p21 which upregulates cyclin E-CDK2, blocking cells from entering the S phase (G1/S blockade). In addition, the cell is prevented from entering the M phase (G2/M blockade) through accumulation of phosphorylated cyclin B-CDK1 complex that results from inactivation cell division cycle 25 (CDC25) C phosphatase by activated Chk2. Ultimately, the cell cycle arrest results in the formation of microenvironment that promotes survival and proliferation of transformed, senescent cells and carcinogenesis [65].

### 2.2. P. gingivalis 

*P. gingivalis* is a major etiological agent in periodontal disease [66,67]. It is an asaccharolytic, non-motile, non-spore forming, short, pleomorphic, gram-negative, black-pigmented, anaerobic rod [68]. It forms a substantial population of the microflora of subgingival sites, buccal mucosa, tongue and tonsillar area in both diseased and healthy individuals [69]. *P. gingivalis* has been shown to present in periodontal pockets of periodontitis patients as well as in healthy individuals [70]. The role of *P. gingivalis* in the development of periodontal disease can be attributed to the many virulence factors that contribute to its defense and destruction against host tissue and epithelial cells. These include fimbriae, hemagglutinin, capsule, lipopolysaccharide, the outer membrane vesicle, and protease gingipains [69,71,72]. The effects of this arsenal of virulence factors extends far beyond the periodontium and the oral cavity, as this species disseminates to distant sites. *P. gingivalis* has been associated with many systemic diseases such as cardiovascular disease, rheumatoid arthritis, preterm birth weight, and diabetes mellitus [73,74]. In addition, studies have shown that periodontitis and *P. gingivalis* are significant risk factors for the development of amyloid-β plaques, dementia and Alzheimer’s disease [75,76,77,78,79]. More recently, post-mortem analysis of brain samples from Alzheimer’s disease patients with periodontitis has shown a genomic fingerprint of *P. gingivalis* along with the protease gingipains localized to the brain [80].

Fimbriae are appendages present on the outer surface of *P. gingivalis* and are involved in cell membrane and crucial to its virulence [68]. *P. gingivalis* fimbriae play a crucial role in nearly all interactions of *P. gingivalis* with not only the host, but also other bacteria. Moreover, fimbriae play a crucial role in *P. gingivalis* adhesion, invasion and colonization of the oral mucosa [69,81]. *P. gingivalis* expresses two types of fimbriae, long and short fimbriae, that are involved in initial attachment and organization of biofilm and attachment to other bacteria [71,82,83]. The long fimbriae, encoded by *FimA* gene, is known as major fimbriae whereas, the short fimbriae, encoded by *Mfa1* gene, is known as minor fimbriae. Interestingly, both fimbriae of *P. gingivalis* have been shown to be important for invasion of dendritic cells (DCs) and induction of differential host immune responses. The minor fimbriae, comprised of a 67-kDa glycoprotein has been shown to be targeting the C-type lectin DC-SIGN on DCs for entry [83,84] and promotion of a pro-survival environment within the DCs [85]. On the other hand, the major fimbriae are composed of a 41 kDa protein called fimbrillin and target toll-like receptor (TLR1) and TLR2 on DCs [86].

The interaction of *P. gingivalis* fimbriae with DCs has been of interest to many researches. More notably, studies on human samples as well as on experimental models, both in vivo and in vitro, have shown that *P. gingivalis* fimbriae play a major role in shaping the host immune response by modulating DCs immune homeostatic functions, mostly favoring *P. gingivalis* invasion and survival within the host. Analysis of CD1c^+^(BDCA-1) CD209^+^ blood myeloid DCs from periodontitis subjects have shown an increased expansion of this DC subset, relative to healthy individuals. In addition, this expansion further increases 24 h after mechanical debridement periodontal plaque and calculus, suggesting a role of bacteremia induced by periodontal pathogens [85]. More interestingly, a study on periodontitis patients with existing coronary artery disease have shown increased myeloid DCs population in systemic circulation. Using postmortem analysis of coronary artery samples of these patients, the study has reported that myeloid DCs are associated with microbial carriage of *P. gingivalis*, where myeloid DCs marker, CD209 (DC-SIGN) shown to co-localize with *P. gingivalis* minor fimbria protein (mfa-1) in the atherosclerotic plaques [85]. The implication of DCs in microbial dissemination of periodontal pathogens has been shown in multiple studies but the mechanism was unclear. Recent studies suggested that *P. gingivalis*, through its minor fimbria via targeting the C-type lectin receptor DC-SIGN, evades the host immune system and manipulates the intracellular signaling pathways in DCs [87]. In addition, it has been shown that the interaction between DC-SIGN receptor on DCs and *P. gingivalis* minor fimbria leads to inhibition of apoptosis and autophagy, protecting the bacteria from antimicrobial clearance and extending survival of *P. gingivalis*-loaded DCs [87]. Autophagy is a process whereby the cell disposes its intracellular damaged proteins and organelles through a lysosome-dependent regulated mechanism by sequestering and directing cargo to the lysosome for degradation. Autophagy is crucial for balancing sources of energy, maintaining proper cellular homeostasis, and defending against invading pathogens [88,89]. Autophagy is involved in many aspects of the host immune response such as clearance of intracellular pathogens by trafficking intracellular pathogens to lysosomes [90,91,92], secretion of inflammatory cytokines [93], antigen presentation [94,95] and development of lymphocytes [96]. Furthermore, autophagy is regulated by a variety of intracellular signaling pathways that are activated in response to the exposure of the pattern recognition receptors (PRRs) of immune cells to ligands or to cytokines. Apoptosis is a programmed cell death that is crucial for elimination of unwanted cells. Apoptosis can be exploited by certain pathogens to extend the survival of host cells [97]. There are two main apoptosis pathways, the intrinsic and the extrinsic pathway. The intrinsic pathway is regulated by intracellular signals that involves B cell lymphoma 2 (BCL-2) family of proteins in the mitochondria. The pro-apoptotic members of BCL-2 family trigger the release of molecules by the mitochondria that stimulates apoptosis process [98]. One of these molecules is cytochrome *c* which plays an important role in the formation of the apoptosome. Apoptosome comprises apoptotic protease-activating factor 1 (APAF1), pro-caspase 9 and cytochrome *c*. Caspase 9 is activated by the apoptosome, which in turn cleaves pro-caspase 3 to form active caspase 3 [99]. On the other hand, the extrinsic pathway is stimulated by external signals that involves binding of the death-inducing factor such as FAS ligand (FASL) to its receptor (FAS) and recruiting the adaptor FAS-associated death domain protein (FADD) and pro-caspase 8, forming death-inducing signaling complex (DISC). Subsequently, pro-caspase 8 is activated in the DISC. The activated caspase 8 then converts pro-caspase 3 to the executioner form, active caspase 3 [100,101,102]. Both the extrinsic and intrinsic pathways converge at the activation of caspase 3 activation, which subsequently cleaves more than 500 cellular substrates to execute the apoptosis process via multiple aspects such as that interfere with transcription, translation, DNA cleavage, cytoskeleton assembly, and membrane trafficking.

**Figure 1:**
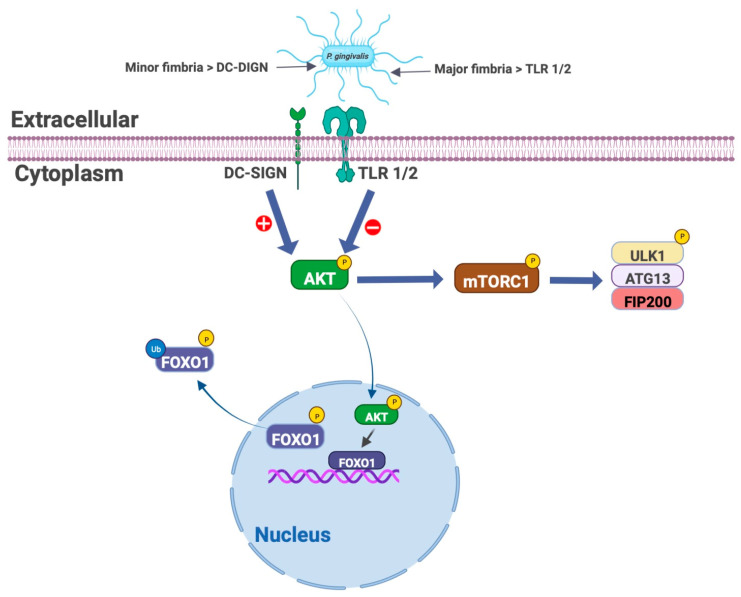
Manipulation of the AKT-FOXO1 pathway and AKT-mTORC1 pathway by *P. gingivalis* fimbriae.

*P. gingivalis* has evolved several immune escape tactics whereby it evades intracellular killing in DCs by targeting DC-SIGN with its minor fimbria and extends the survival of the host DCs to live in [90]. The uptake of *P. gingivalis* by DCs via DC-SIGN-dependent manner results in a decrease in the intracellular killing and an increase in the intracellular content of *P. gingivalis* inside DCs. Autophagy is regulated by the AKT-mTOR signaling axis which regulates the cell survival mechanisms through mTOR-dependent autophagy during physiologic as well as pathologic conditions. The inhibition of the AKT-mTOR pathway in DCs is one of the strategies of *P. gingivalis* to survive inside DCs and evade the host immune response [87], where *P. gingivalis* infection increases expression of the important downstream elements involved in this pathway such as p-Akt Ser473, p-mTOR Ser2448, p-Raptor Ser792 and p-ULK1 Ser757 [87]. It is noteworthy that blocking the receptor DC-SIGN on DCs by HIV glycoprotein 120 results in reduction of survival of *P. gingivalis* inside DCs [90]. Furthermore, *P. gingivalis* minor fimbria induces dysregulation of apoptosis in DCs. In addition to its role in regulating autophagy, the AKT pathway inhibits apoptosis via translocation of phosphorylated AKT from the cytoplasm to the nucleus, where it subsequently phosphorylates FOXO1, leading to translocation of phosphorylated FOXO1 to the cytoplasm, where it undergoes polyubiquitination and lysosomal degradation [103] (Figure 1). As a result, apoptosis is inhibited. Surprisingly, targeting the receptor DC-SIGN on DCs by *P. gingivalis* minor fimbria results in the activation of the AKT-FOXO1 pathway in addition to upregulation of the expression of pro-apoptotic protein BCL2 and downregulation of BIM, BAX and cleaved caspase 3 expression [87]. Extended survival of *P. gingivalis*-loaded DCs might be contributing to the systemic inflammation and dissemination of *P. gingivalis* to distant sites, which could be a result of *P. gingivalis* exploiting DCs migratory functions, with impaired pathogen clearance and extended survival. Inhibition of the host cell programmed cell death is the same tactic that *P. gingivalis* uses to exploit gingival epithelial cells for survival within, via manipulation of the JAK-Stat pathway [32]. The influence of *P. gingivalis* on the modulation of the host cells apoptotic cell death has been reported on a variety of cells, including immune cells, fibroblasts, epithelial cells and endothelial cells [104,105,106,107,108,109]. 

Abnormal survival of immune cells can lead to dire consequences such as development of autoimmune diseases and cancer [105]. *P. gingivalis* has been reported to promote generation of myeloid-derived suppressor cells (MDSCs), pathologically activated neutrophils and monocytes with potent immunosuppressive activity. Consistent with the immunosuppressive role of MDSCs is the ability of *P. gingivalis*-generated MDSCs to inhibit CD8+ T cells while induce FOXP3 + T_regs_ through the anti-apoptotic pathway AKT-FOXO1 [110]. In addition, certain intracellular signaling pathways crucial for regulation of apoptosis has been reported to be influenced by *P. gingivalis* differential fimbria expression, leading to promotion proliferation of oral squamous cell carcinomas (OSCCs) [110]. Altogether, these studies highlight the implication of *P. gingivalis* fimbria in the induction of immunosuppression and oncogenic cell proliferation, suggesting implication of *P. gingivalis* in the prognosis of oral cancers in patients with periodontitis. 

Gingipains are cysteine endopeptidases that play an essential role in the pathogenicity of *P. gingivalis* in periodontal disease. They are expressed and located on the outer membranes of *P. gingivalis* or secreted into the extracellular environment [111]. There are two types of gingipains, arginine-specific protease (Rgp; encoded by *rgpA* and *rgpB*) and lysine-specific protease (Kgp; encoded by *kgp*). In addition to their function as a proteolytic tool for the degradation of proteinaceous nutrients to *P. gingivalis* for growth, gingipains are essential for the processing of fimbrial proteins to facilitate bacterial attachment and adhesion to the host [112,113]. In addition, gingipain can facilitate bacterial evasion of the host immune response by cleaving surface receptors and cytokines degradation [114]. Gingipain has been shown to promote cellular invasion and metastasis of OSCC cells via activation of the ERK1/2-Ets1, p38/HSP27, and PAR2/NFκB pathways to induce proMMP9 expression [115]. Recently, a study has reported that wild-type *P. gingivalis* 33,277 can promote colorectal cancer cell proliferation via activation of the MAPK/ERK signaling pathway, comparing to the gingipain-deficient mutant KDP136, suggesting an important role of gingipain in colorectal cancer [116].

### 2.3. F. nucleatum

**Figure 2:**
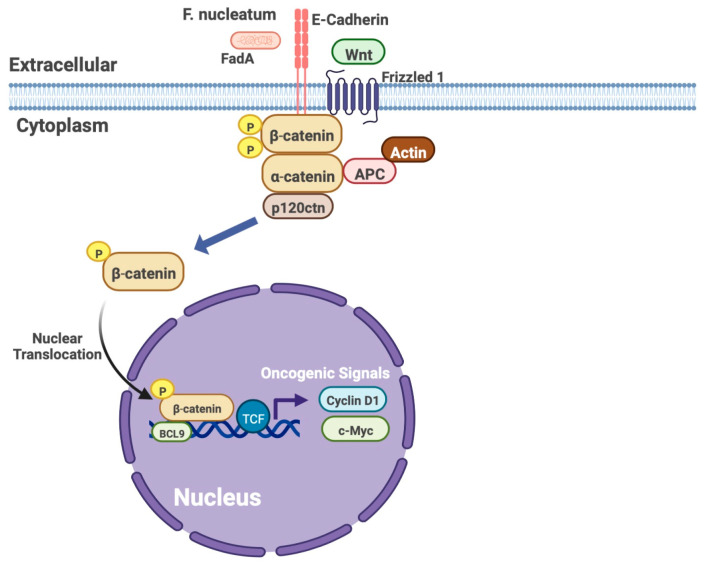
Induction of carcinogenesis by *F. nucleatum* FadA via modulation of Wnt/β-catenin signaling pathway.

*F. nucleatum* is a Gram-negative anaerobic filamentous spindle-shaped rod. Unlike other strict anaerobic bacteria, *F. nucleatum* possess NADH oxidase endowing them with a limited ability to survive in oxygenated environment [117]. In addition to its implication in periodontal disease [118], *F. nucleatum* is capable of systemic dissemination and causing extra-oral infections, such as brain, liver, spleen, and lung abscesses, septicemia related infections, pelvic inflammatory disease, and intrauterine infections [119,120,121,122,123,124]. *F. nucleatum* is equipped with a variety of adhesins that enable it to adhere to various microorganisms. These adhesion proteins are considered the main virulence factors. Among all the adhesins expressed by *F. nucleatum*, only Fusobacterium adhesin A (FadA), has been identified to be capable of binding to host cells. FadA exists in two forms; non-secreted, intact pre-FadA and secreted, mature FadA (mFadA). pre-FadA is anchored to the to the inner membrane of *F. nucleatum* while mFadA is secreted outside of the bacterium [125]. Pre-FadA and mFadA together form a complex called FadAc that is required for *F. nucleatum* attachment and invasion of the host cells. FadA binds to cadherin family receptors, mainly E-cadherin and vascular endothelial (VE) cadherin (CDH5), for adhesion and invasion of the host [126]. Binding of FadA to E-cadherin on epithelial cells results in phosphorylation and internalization of E-cadherin and the activation of the canonical Wnt pathway, one of the key signaling cascades regulating development and stemness, and has also been tightly associated with promotion of carcinogenesis. Furthermore, FadA binds to VE-cadherin on vascular endothelial cells, resulting in migration of the endothelial cells, increasing endothelial permeability. Therefore, FadA plays a role not only in the invasion of host cells but also allow in microbial dissemination to blood circulation by increasing endothelial permeability, contributing to spread of infection and immune escape [126]. Modulation of E-cadherin/β-catenin signaling by FadA has been implicated in the promotion of colorectal carcinogenesis [127] (Figure 2).

**Figure 3:**
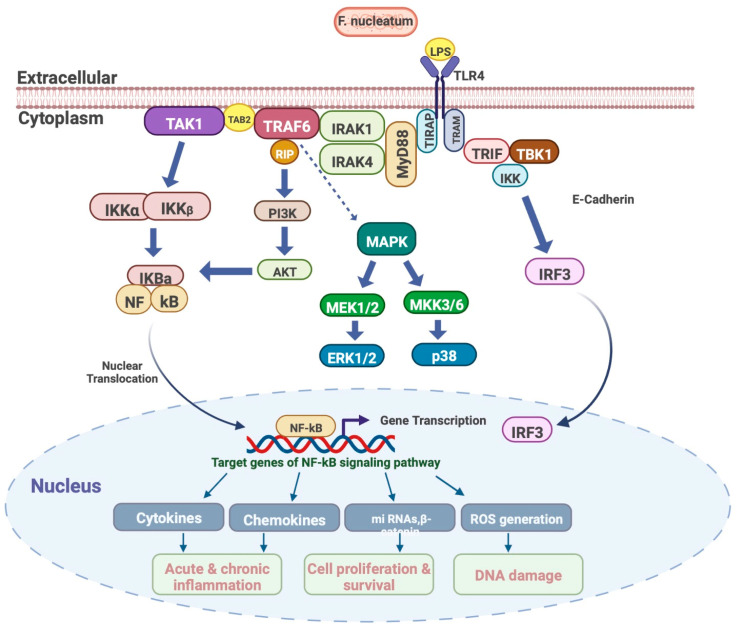
Targeting NF-κB signaling pathway by *F. nucleatum* LPS.

*F. nucleatum* also expresses an outer membrane protein, familial adenomatous polyposis 2 (Fap2), that binds to the inhibitory receptor T cell immunoreceptor with Ig and ITIM domains (TIGIT), that is expressed by human natural killer (NK) cells and lymphocytes. Hence, Fap2 influences the NK cells and lymphocytes by suppressing the cytotoxic activities, ultimately facilitating evasion of the host immune system by tumor cells and promoting the formation of inflammatory microenvironment [128]. Furthermore, LPS of *F. nucleatum* binds to TLR4 on the host cells and interact with Toll/IL-1 receptor (TIR) [129], resulting in the recruitment of myeloid differentiation primary response protein 88 (MyD88), which in turn induces phosphorylation of IL-1 receptor–associated kinase (IRAK). Subsequently, IRAK dissociates from the receptor and interacts with adaptor proteins TNFR-associated factor 6 (TRAF6) and TAK1 -binding proteins 2 (TAB2) on the cell membrane. TRAF6 becomes targeted for ubiquitination (Ub) and activates TGF-β-activated kinase 1 (TAK1) and TAB2/3, resulting in the activation of I-κB (IκB) and mitogen-activated protein kinase (MAPK). Activated IκB and MAPK induce subsequent translocation of nuclear factor-κB (NF-κB) and AP-1 to the nucleus [130]. NF-κB is involved in the induction of the expression of many genes, including genes encoding pro-inflammatory cytokines and chemokines, and inflammasome regulation. In addition, NF-κB plays an important role in regulating the survival, activation and differentiation of immune cells. Of particular interest, this signaling pathway is involved in promoting cell proliferation and closely related to cancer development and progression, of which is promotion of proliferation of pancreatic ductal adenocarcinoma (PDAC) and colorectal cancer [129,131] (Figure 3). 

## 3. Conclusions

Periodontal pathogens are equipped with an arsenal of virulence factors. Some of these factors are attached to the outer membrane of the bacteria and others are secreted in the inflammatory milieu. These virulence factors play a significant role in the invasion of the host cells, secretion of inflammatory cytokines and chemokines, and pathogen dissemination to the blood stream and to distant sites. Some periodontal pathogens have evolved immune escape tactics the involve not only protecting them from antimicrobial killing inside host cells, but also extending the survival of such cells and exploiting their migratory profile to hitch-hike to distant sites. Most of these immune deregulation events are the result of the modulation of intracellular signaling pathway that is influenced by the interaction between the virulence factors and immune-receptors of the immune cells. In addition, some of the affected signaling pathways are suggested to be implicated in the promotion of carcinogenesis. Most of the studies that reported the association of periodontal disease with different types of cancer are based on cross-sectional studies, reporting elevated tumor markers in these individuals. Furthermore, in vitro studies are still the only tools that dissected the mechanism through which some periodontal pathogens promote specific types of cancer (Table 1). Interestingly, it is still unknown why some patients with periodontal disease develop cancer and this aspect should be examined in future studies.

## Figures and Tables

**Table 1:** List of different types of cancer associated with periodontal disease and the proposed pathomechanisms.

Cancer Type	Proposed Pathomechanism
Pancreatic cancer	Activation of Akt signaling pathway [132] Modulation of NF-κB pathway [129,131]
Head and neck SCC	Activation of AKT-FOXO1 pathway [110] Activation of ERK1/2-Ets1, p38/HSP27, and PAR2/NFκB pathways [115] Regulation of ATR and NLRP3 Inflammasome [133]
Prostate cancer	Modulation of NOD1/NOD2 signaling pathway [134]
Colorectal cancer	Activation of the MAPK/ERK pathway [116] Modulation of Wnt/β-catenin signaling pathway [127]

## Data Availability

Not applicable.
